# Correction: Assessing the feasibility of an integrated collection of education modules for fall and fracture prevention (iCARE) for healthcare providers in long term care: A longitudinal study

**DOI:** 10.1371/journal.pgph.0004933

**Published:** 2025-07-15

**Authors:** The PLOS Global Public Health Editors

## Notice of Republication

This article [1] was republished on 1 July 2025, to address an issue identified post-publication. Please download this article again to view the correct version.
