# Supplementary material for: Assessing the feasibility of an integrated collection of education modules for fall and fracture prevention (iCARE) for healthcare providers in long term care: A longitudinal study
Source: PLOS Glob Public Health. 2024 Nov 25;4(11):e0003096. doi: 10.1371/journal.pgph.0003096 (PMC11588276; doi:10.1371/journal.pgph.0003096)
Supplement: S1 File — (PDF) [file pgph.0003096.s001.pdf]

PREVENT Components

The PREVENT model includes several components including train-the-trainer, educational manuals and other resources, audit and feedback report, care planning, and academic detailing.

Train-the-trainer: The focus of the train-the-trainer workshop was to guide the implementation process and promote best practices after the research team has left. The lead investigator (IBR) met with the local champion over three sessions to review the education materials and planning tools. During the first session, the local champion was provided with a Fracture and Falls handbook which contains information about PREVENT and links to varies studies and toolkits. During the second session we met with the local champion and the pharmacist reviewed how to deliver the educational module and evidence-based methods to develop an effective Audit and Feedback report (18). During the third session, the local champion met with the research team to practice delivering the educational module and review any questions about fracture and fall management strategies.

Educational manual and point of care tools: In collaboration with healthcare professionals in LTC, we developed an evidence-based fall and fractures prevention manual for healthcare professionals. The purpose of the manual was to design a tool that contained all the evidence-based information and practical tips in one location. The manual was divided into sections dedicated to specific types of healthcare professionals; for example, the “recommendations for physicians & pharmacists” section provided a one-page guide to prescribing osteoporosis medications and contraindications. In addition, each section contained practical tips on how to implement the evidence-based recommendation. Practical tips included a checklist of steps, corresponding images, short online videos, point of care tools, and QR codes or web links to peer-reviewed publications. The Fall and Fracture Prevention Manual went through several iterations to ensure the information was accurate and practical. The material was written in accordance with average national literacy levels and reviewed by healthcare providers on the research team.

Educational Module: The local champion delivered a one-hour educational module to the leadership team. The educational meeting used a combination of a didactic presentation and interactive activities to identify high risk of fracture individuals and review best practices for

osteoporosis management. In addition, we offered a continuing medical education (CME) work 1 MainPro credit to physicians. The CME was delivered by two physicians (family physician and geriatrician) with expertise in osteoporosis management. Two continuing medical education modules were offered to physicians over the course of the study. The first event was on February 17th, 2023, and the second on March 31st, 2023. One physician and no members from the leadership team from Home A attended the first module. The attendance rate increased at the second module with one physician and eight members of the leadership team at Home B and two members of LTC Home C's leadership team attending this educational event.

Audit and Feedback: The Audit and Feedback report was presented during the educational meeting and created based on chart abstraction. The reports provided homes with information about the percentage of residents at high versus low risk of fracture, the type of fractures sustained in their home, and the number of residents on osteoporosis medications.

Implementation Intention: After the educational meeting, the leadership team engaged in implementation intentions guided by the "plan-do-study-act" cycle. The cycle approach was developed in the field of Continuous Quality Improvement and considers three key questions based on the community of practice model: 1) what is to be accomplished, 2) how we know that a change is an improvement, and 3) what changes can we make that will result in an improvement. The plan-do-study-act process engaged the internal champion and the leadership team in planning by breaking goals into manageable chunks, testing ideas, and assessing the results in order to better monitor the impact of changes. During action planning, the internal champion and the leadership team brainstorm facilitators and barriers to identifying fracture risk scores among residents and providing fracture prevention care to residents at high risk of fracture.

Care planning: The care plans of residents at high risk were guided by the fracture prevention care planning recommendations in addition to receiving their usual care. Recommendations include diet and supplement (i.e., consuming 1200 mg/day of dietary calcium and additional 500 mg/day of calcium supplements if diet is not reached, and 800-2000 IU/day of vitamin D supplementation), multifactorial fall and fracture prevention (i.e., hip protectors for those who are mobile, exercise prescriptions, medication review, assessment of environmental hazards, review of assistive devices, and management of urinary incontinence), and medications (i.e., medication review).

Academic detailing: The goal of academic detailing is to improve clinician decision-making through unbiased information to enhance evidence-based care and improve resident's outcomes. Academic detailing was offered by the consultant pharmacist or by the physicians on the research team. Clinicians would email their questions about osteoporosis management to our academic detailer who would provide concise decision support material to further encourage evidence-based clinical decisions.

References

1. Zimmerman S, Girman C, Buie V, Chandler J, Hawkes W, Martin A, et al. The Prevalence of Osteoporosis in Nursing Home Residents. Osteoporos Int. 1999;9:151–7.

2. Neuman M, Silber J, Magaziner J, Passarella M, Mehta S, Werner R. Survival and functional outcomes after hip fracture among nursing home residents. JAMA Intern Med. 2014;174(8):1273–80.

3. Van Den Bergh JP, Van Geel TA, Geusens PP. Osteoporosis, frailty and fracture: Implications for case finding and therapy. Nat Rev Rheumatol [Internet]. 2012;8(3):163–72. Available from: http://dx.doi.org/10.1038/nrrheum.2011.217

4. Ioannidis G, O'Donnell D, Kennedy C, Navare H, Giangregorio L, Cheung A, et al. What triggers osteoporosis therapy in high-risk residents living in long-term care homes?The Gaining Optimal Osteoporosis Assessments in Long-Term Care (GOAL) study. In: WCO-IOF-ESCEO World Congress on Osteoporosis, Osteoarthritis and musculoskeletal diseases. p. 26 S52.

5. Papaioannou A, Santesso N, Morin SN, Feldman S, Adachi JD, Crilly R, et al. Recommendations for preventing fracture in long-term care. CMAJ. 2015;187(15):1135–44.

6. RNAO. Reports on Long-Term Care Homes [Internet]. 2007 [cited 2021 Dec 27]. Available from: http://publicreporting.ltchomes.net/en-ca/default.aspx

7. Papaioannou A, Ioannidis G, McArthur C, Hillier LM, Feldman S, Giangregorio L, et al. Preventing Fractures in Long-Term Care: Translating Recommendations to Clinical Practice. J Am Med Dir Assoc [Internet]. 2021;22(1):36–42. Available from: https://doi.org/10.1016/j.jamda.2020.07.003

8. Giangregorio L, Jantzi M, Papaioannou A, Hirdes J, Maxewell C, Poss J. Osteoporosis management among residents living in long-term care. Osteoporos Int. 2009;20(9):1471–8.

9. Sawka A, Ismaila N, Raina P, Thabane L, Straus S, Adachi J, et al. Hip fracture prevention strategies in long-term care: A survey of Canadian physicians' opinions. Can Fam Physician. 2010;56:e392-397.

10. Graham ID, Tetroe J, Gagnon M. Knowledge Translation in Healthcare. Second Edi. Sons JW&, editor. 2013. 75–92 p.

11. Sibley LM, Weiner J. An evaluation of access to health care services along the rural-urban continuum in Canada. BMC Heal Serv Res. 2011;11(20):1–11.

12. Mahler H, Fleisig R. MIRA innovation workbook: User-centred reserach. 2017.

13. Michie S, Richardson M, Johnston M, Abraham C, Francis J, Hardeman W, et al. The behavior change technique taxonomy (v1) of 93 hierarchically clustered techniques: Building an international consensus for the reporting of behavior change interventions. Ann Behav Med. 2013;46(1):81–95.

14. Intersectinality & Knowledge Translation: Guide for common appraoches to assessing barriers and facilitators to knowledge use [Internet]. Available from: https://knowledgetranslation.net/wp-content/uploads/2020/02/IntersectionalityKT_Guide_for_Common_Approaches_to_Assessing_Barriers_and_Facilitators_Draft_Version.pdf

15. Glasgow R, Vogt T, Boles S. Evaluating the public health impact of health promotion interventions: the RE-AIM framework. AM J Public Heal. 1999;89(9):1322–7.

16. Joven M, Tung E, Bell D. Barriers to Osteoporosis Treatment in the Nursing Home. JAMDA. 2016;17(3).

17. McArthur C, Bai Y, Hewston P, Giangregorio L, Straus S, Papaioannou A. Barriers and facilitators to implementing evidence-based guidelines in long-term care: a qualitative evidence synthesis. Implement Sci. 2021;16(1).

18. Brehaut JC, Colquhoun HL, Eva KW, Carroll K, Sales A, Michie S, et al. Practice feedback interventions: 15 suggestions for optimizing effectiveness. Ann Intern Med. 2016;164(6):435–41.

19. Kluger A, Van Dijk D. Feedback, the various tasks of the doctor, and the feedforward alternative. Med Educ. 2010;44:1166–74.
