# Supplementary material for: Assessing the feasibility of an integrated collection of education modules for fall and fracture prevention (iCARE) for healthcare providers in long term care: A longitudinal study
Source: PLOS Glob Public Health. 2024 Nov 25;4(11):e0003096. doi: 10.1371/journal.pgph.0003096 (PMC11588276; doi:10.1371/journal.pgph.0003096)
Supplement: S1 Table — (PDF) [file pgph.0003096.s003.pdf]

STROBE Statement—Checklist of items that should be included in reports of ***cohort-studies***

	Item No	Recommendation	Respected?	Comments and Quotes
**Title and abstract**	1	(*a*) Indicate the study's design with a commonly used term in the title or the abstract	Yes	"The iCARE feasibility non-experimental design study: An integrated collection of education modules for fall and fracture prevention for healthcare providers in long term care." [pg. 1 L1]
		(*b*) Provide in the abstract an informative and balanced summary of what was done and what was found	Yes	These information are stated in the study abstract (study objective described, method and results described)
**Introduction**				
Background/rationale	2	Explain the scientific background and rationale for the investigation being reported	Yes	Scientific background and rationale are provided in the Introduction section [pg. 1-2 L53-84]
Objectives	3	State specific objectives, including any prespecified hypotheses	Yes	The study objectives are found in the last paragraph of the Introduction section<br>"The purpose of the iCare (integrated collection of education modules for fall and fracture prevention) study was to determine the feasibility of implementing PREVENT to healthcare providers in LTC. Our secondary outcomes were to determine if PREVENT improved knowledge uptake of the Canadian recommendations (9) and increased the proportion of osteoporosis medication prescriptions post-intervention." [pg. 4 L88-92]
**Methods**				
Study design	4	Present key elements of study design early in the paper	Yes	Study design is stated in the first subsection of Methods.<br>"We conducted a pre-post non-experimental design study in three LTC homes across Ontario." [pg. 4 L98-99]

				All key study elements are described in the Methods section.
Setting	5	Describe the setting, locations, and relevant dates, including periods of recruitment, exposure, follow-up, and data collection	Yes	The setting, locations, and dates for recruitment and implementation of the intervention are described in sections "Study Setting" [pg. 4], "Knowledge Translation Model" [pg. 5-6] and "Intervention Timeline" [pg. 7]. Locations were de-identified to protect the anonymity of the participants.
Participants	6	(*a*) Give the eligibility criteria, and the sources and methods of selection of participants. Describe methods of follow-up	Yes	The eligibility criteria can be found under the "Inclusion/Exclusion Criteria" subsection of the Methods section [pg. 7 L153-157]<br>The study population is described the "Study Setting" section [pg. 4] and methods for identifying specific participants in each location are discussed in the "Knowledge Translation Model" section. [pg. 7]
		(*b*) For matched studies, give matching criteria and number of exposed and unexposed	N/A	
Variables	7	Clearly define all outcomes, exposures, predictors, potential confounders, and effect modifiers. Give diagnostic criteria, if applicable	Yes	The primary and secondary outcomes of the study are outlined in the Methods section under subsection "Outcomes" [pg. 7-9 L159-196]
Data sources/ measurement	8*	For each variable of interest, give sources of data and details of methods of assessment (measurement). Describe comparability of assessment methods if there is more than one group	Yes	Recruitment rates and fidelity scores were assessed using descriptive statistics. [pg. 9 L198-199]<br>Focus groups were analysed using content analysis and data was coded using NVivo, version 14. [pg. 9 L199-201]<br>Change in knowledge uptake was assessed using a paired sample t-test [pg. 9 L205-206]<br>Changes in osteoporosis medications were determined from the absolute change from pre-

				post intervention prescriptions. [pg. 9 L206-209]
Bias	9	Describe any efforts to address potential sources of bias	No	Potential sources of bias were not addressed in this study.
Study size	10	Explain how the study size was arrived at	No	
Quantitative variables	11	Explain how quantitative variables were handled in the analyses. If applicable, describe which groupings were chosen and why	Yes	The analysis of quantitative variables were stated in the “Analysis” section. A description outlining the reasoning for analysing osteoporosis medications is provided on page 4 lines 92-95.<br>“To report changes in osteoporosis medications, we conducted two data pulls at baseline and post-intervention; we included residents who “moved-in” (i.e., moved into the home during the post-intervention phase) and “moved-out” (i.e., passed away during the post-intervention phase) in the analysis. We reported the results using absolute change. Quantitative results were analysed using IBM SPSS Statistics for Windows, version 28 (Armonk, NY: IBM Corp).” [pg. 9 L206-211]
Statistical methods	12	(*a*) Describe all statistical methods, including those used to control for confounding	Yes	Descriptive statistics were used to report recruitment rates and fidelity scores as a total score or value. [pg. 9 L198-99]<br>A paired sample t-test was used to assess change in knowledge uptake and all quantitative results were analysed using IMB SPSS Statistics for Windows, version 28. [pg. 9 L205-211]
		(*b*) Describe any methods used to examine subgroups and interactions	No	No subgroup analyses were done.
		(*c*) Explain how missing data were addressed	N/A	There are no missing data for this study.
		(*d*) If applicable, explain how loss to follow-up was addressed	N/A	
		(*e*) Describe any sensitivity analyses	N/A	No sensitivity analyses were done.
**Results**				
Participants	13*	(a) Report numbers of individuals at each stage of study—eg numbers		Knowledge of how many homes (N=3)a nd

		potentially eligible, examined for eligibility, confirmed eligible, included in the study, completing follow-up, and analysed		healthcare providers from each home (n=10,19,11, respectively) are reported under the “Feasibility” subsection of the Results section [pg. 9-11] and in Table 3.<br>“Table 3: Local champion and leadership team characteristics (n = 3)” [pg. 10 L228]
		(b) Give reasons for non-participation at each stage		Study recruitment was affected due to the COVID-19 pandemic, and therefore we did not achieve our intended sample size of five homes and recruited three homes.<br>“We recruited three LTC homes between October 2022 and February 2023; our recruitment process was affected by residual COVID-19 complications.” [pg. 9 L214-215]
		(c) Consider use of a flow diagram	N/A	
Descriptive data	14*	(a) Give characteristics of study participants (eg demographic, clinical, social) and information on exposures and potential confounders	Yes	Demographic characteristics of study participants are found under subsection “Feasibility” of the Results section [pg. 9-11] and in Table 3. [pg. 10 L228]
		(b) Indicate number of participants with missing data for each variable of interest	N/A	
		(c) Summarise follow-up time (e.g., average and total amount)	N/A	
Outcome data	15*	Report numbers of outcome events or summary measures over time	Yes	Quantitative results including feasibility (fidelity scores) [pg. 11 L231-233], knowledge uptake [pg. 13 L309-312], and osteoporosis medications [pg. pg.13-14 L313-321, including Table 4] were reported under the “Feasibility” and “Audit Report” subsections of the results section.<br>Qualitative results from the focus groups were reported in the Results section under subsection “Challenges, successes, and adaptations”

Main results	16	(*a*) Give unadjusted estimates and, if applicable, confounder-adjusted estimates and their precision (eg, 95% confidence interval). Make clear which confounders were adjusted for and why they were included	Yes	95% confidence intervals were reported under subsection "Audit Report" of the Results section for assessing change in knowledge uptake and change in osteoporosis medications. [pg. 13-14 L312-316] Odds ratios were reported in addition to the 95% confidence intervals for osteoporosis medications.
		(*b*) Report category boundaries when continuous variables were categorized	N/A	
		(*c*) If relevant, consider translating estimates of relative risk into absolute risk for a meaningful time period	N/A	
Other analyses	17	Report other analyses done—eg analyses of subgroups and interactions, and sensitivity analyses	N/A	
**Discussion**				
Key results	18	Summarise key results with reference to study objectives	Yes	Key results are throughout the Discussion section [pg. 14-15 L323-346], and are also summarized in the Conclusion section. [pg. 17-18]
Limitations	19	Discuss limitations of the study, taking into account sources of potential bias or imprecision. Discuss both direction and magnitude of any potential bias	Yes	Description of limitations is done in the fifth paragraph of the Discussion section. [pg. 17 L408-418]
Interpretation	20	Give a cautious overall interpretation of results considering objectives, limitations, multiplicity of analyses, results from similar studies, and other relevant evidence	Yes	References were added where possible, and discussed. Limitations were taken into account in the discussion.
Generalisability	21	Discuss the generalisability (external validity) of the study results	Yes	"The sustainability or scalability of this model may not be applicable in the Canada healthcare system. Thus, the next steps will be to conduct a large randomized controlled trial of the adapted PREVENT model in several Canadian LTC homes to determine if the model reduces the rate of hip fractures in residents at high risk of fracture." [pg. 16 L378-381]

**Other information**				
Funding	22	Give the source of funding and the role of the funders for the present study and, if applicable, for the original study on which the present article is based		Funding information is displayed on page 23 lines 595-598.

*Give information separately for cases and controls.

Note: An Explanation and Elaboration article discusses each checklist item and gives methodological background and published examples of transparent reporting. The STROBE checklist is best used in conjunction with this article (freely available on the Web sites of PLoS Medicine at http://www.plosmedicine.org/, Annals of Internal Medicine at http://www.annals.org/, and Epidemiology at http://www.epidem.com/). Information on the STROBE Initiative is available at http://www.strobe-statement.org.
