# Supplementary material for: Assessing the feasibility of an integrated collection of education modules for fall and fracture prevention (iCARE) for healthcare providers in long term care: A longitudinal study
Source: PLOS Glob Public Health. 2024 Nov 25;4(11):e0003096. doi: 10.1371/journal.pgph.0003096 (PMC11588276; doi:10.1371/journal.pgph.0003096)
Supplement: S4 Table — (PDF) [file pgph.0003096.s006.pdf]

S4 Table: Feasibility outcomes results

Recruitment rate (organizational level – length of time to recruit three LTC homes)	5 months
Recruitment rate (participant level - length of time to identify local champion)	1 month
Recruitment rate (participant level - length of time for local champion to review educational materials, adapt material to the LTC home, and develop audit and feedback report)	2 months
Recruitment rate (summary - length of time to recruit a LTC home, train the local champion and implement the PREVENT model)	10 to 12 months
