# Supplementary material for: Assessing the feasibility of an integrated collection of education modules for fall and fracture prevention (iCARE) for healthcare providers in long term care: A longitudinal study
Source: PLOS Glob Public Health. 2024 Nov 25;4(11):e0003096. doi: 10.1371/journal.pgph.0003096 (PMC11588276; doi:10.1371/journal.pgph.0003096)
Supplement: S5 Table — (PDF) [file pgph.0003096.s007.pdf]

S5 Table: Full list of themes arising from the content analysis. Themes are shown with illustrative quotes.

Themes	Quote
**Challenges**<br>• Unclear sustainability strategy<br>• Misperceptions between fall and fracture prevention strategies<br>• Fear of prescribing osteoporosis medications<br>• Time barriers with completing the audit report	"I found myself often sometimes ignoring the prompts for FRS unless there was a specific reason. Cold calling families to say your fracture risk scale is high, and that's the only reason I'm calling is weird." [Home B]<br><br>"I think a lot of homes already are looking at all the PREVENT aspects, it's just not known as PREVENT or they're not grouping them together, but I think it's just something that's already being looked at from all the different disciplines." [Home A]<br><br>"It's worked well just as a tool to let us know if there's any missing boxes that we're not checking for fracture prevention. It's very helpful with just making sure we're looking at everything and yea, just crossing off any things we can possibly to do help the residents." [Home A]<br><br>"We are worried that, we weren't overly confident that would lead to prescription. So, there was a trigger. We didn't know if they were going to have informed consent and we didn't know if our nursing staff knew enough about these medications to provide it" [Home B].
**Facilitating Factors**<br>• Easy access to the FRS<br>• Clear and succinct educational material tailored to each healthcare professional<br>• Educational module with an accredited program for physicians and nurses	"A visual to see the walkers and those pieces, I think even the slide with the hip protectors, like how to apply them and which ones were incorrect. I think that's a nice reminder because I didn't even know how to position them correctly and I didn't even have awareness of the FRS. Like I kind of knew like our FRATscore and our falls risk, and when I go to my units I know who the falls, the higher risk of falls are but I didn't, and then I just thought of them like okay, we have hip protectors for them, like what are we doing about it but I didn't necessarily do a full review of like let's look at their meds and let's look at their hips, are they wearing hip protectors, what are they, let's look at all these other things. I wasn't sort of looking at that." [Home C].

**Adaptations**<br>• Identifying additional characteristics for a successful local champion<br>• Developing an automated audit report<br>• Creating tools to facilitate conversations for prescribing osteoporosis medications	"… once everyone gets used to that new documentation I think they will adjust to the fact that we have an FRS and also a FRAT because resident care managers are mostly concerned about the FRAT score whereas now that we've just changed the falls, then the nurses that are completing those assessments will be focused on both instead of just the one." [Home B]

FRS = Fracture Risk Scale; FRAT = Falls Risk Assessment Tool
