# Supplementary material for: Assessing the feasibility of an integrated collection of education modules for fall and fracture prevention (iCARE) for healthcare providers in long term care: A longitudinal study
Source: PLOS Glob Public Health. 2024 Nov 25;4(11):e0003096. doi: 10.1371/journal.pgph.0003096 (PMC11588276; doi:10.1371/journal.pgph.0003096)
Supplement: Attachment — Submitted filename: Response to reviewers_iCARE Manuscript.docx [file pgph.0003096.s008.docx]

Drs. Catherin Kyobutungi and Dr. Madhukar Pai

Editors-in-Chief

PLOS Global Public Health

August 13, 2024

Dear Drs. Kyobutungi and Pai,

Thank you for reviewing our manuscript: “The iCARE Study: An integrated collection of education modules for fall and fracture prevention for healthcare providers in long term care”. We have addressed your comments below. The line and page numbers are associated with the Clean version of the manuscript. Should you have any questions, please do not hesitate to contact me ([papaioannou@hhsc.ca](mailto:papaioannou@hhsc.ca)).

Yours sincerely,

Dr. Alexandra Papaioannou, BScN, MD, MSc, FRCP(C), FACP, FCGS

Professor of Medicine, Divisions of Geriatric, McMaster University

Medicine & Rheumatology and Department of HEI

Tier 1 Canada Research Chair in Geriatric Medicine & Healthy Aging

Executive Director, Geras Centre for Aging Research

Chair, Regional Geriatric Program (RGP) Central

Thank you for reviewing our manuscript: “The iCARE Study: An integrated collection of education modules for fall and fracture prevention for healthcare providers in long term care” (new title: “Assessing the feasibility of an integrated collection of education modules for fall and fracture prevention (iCARE) for healthcare providers in long term care: A longitudinal study”. We have addressed your comments below. The line and page numbers are associated with the clean version of the manuscript.

**Reviewer #1**: I enjoyed reading the article, and I commend the attention to detail. The authors aimed to determine the feasibility of implementing the PREVENT model in long-term care homes, focusing on falls and hip fractures. Proposed or reported benefits of the model include easy access to the Fracture Risk Scale, clear educational material, and an accredited educational module. Challenges included misperceptions, fear of side effects, and time barriers. The study did not increase knowledge uptake of the guidelines.

1. In the study setting, you noted that 2 out of 5 LTC agreed, yet you worked with 3. How did you achieve this?

We approached five LTC organizations; however, only two organizations were interested in participating in the study. One organization provided us with one home, while the second organization provided us with two homes. We have clarified this point in the study setting: “*Between January to June 2022, we sent emails to five organizations operating several LTC homes in Ontario; only two organizations were interested in participating in our study. One organization recruited one LTC home, while the second organization recruited two homes. We recruited one for-profit and two not-for profit homes, herein known as Home A, 120 beds, Home B, 425 beds, and Home C,240 beds. All three homes are located in southern Ontario, Canada in large metropolitan cities.*” (page 4, lines 109-114)

2. Comparing the preaudit and post-audit reports, changes appear to be minimal; a p value could have helped identify if the changes were at least statistically significant.

We conducted a McNemar’s test on the following outcomes: number and percentage of residents at high risk of fracture, number and percentage of residents who sustained a fragility fracture in the last six months, and number and percentage of residents at high risk of fracture on osteoporosis medications (unspecified amount of time). We added p-values to identify changes from pre-to-post audit reports as found in the audit report section of the results: “*The results of the audit report are shown in Table 4. Home A results indicated the number and percentage of residents at high risk of fracture on fracture prevention medications increased by 16.5% (pre= 19/97 = 19.6% versus post= 35/97 = 36.1%; p-value = 0.005). Non-statistically significant results were found for the number of residents at high risk of fracture changed by 6.2% (pre = 52/97 = 53.6% versus post = 58/97 = 59.8%; p-value = 0.41) and the number and percentage of residents who sustained a fragility fracture in the last six months did not change (pre = 3/97 = 3.1% versus post = 3/97 = 3.1%; p-value = 1.0).*” (page 14, lines 329-335). Given the data were aggregated in Homes B and C, we are unable to perform this test for these two homes and results are reported by absolute change: “*Home B reported an absolute change of 3% (31% at baseline and 34% post-intervention), while Home C increased by 11% (26% at baseline and 37% post-intervention).*” (page 14, lines 334-342)

3. Setting the stage for a much larger project, the author might wish to add a recommendation section on what can be done to improve the efficacy of PREVENT in reducing falls and hip fractures rather than mentioning it in passing.

The purpose of the iCare study was to determine the feasibility of implementing the PREVENT model. At this time, we are unable to comment on strategies to improve the effectiveness of PREVENT in reducing hip fractures as this data was not obtained in the iCare study. Next steps will be to conduct a large, pragmatic, randomized controlled trial to assess the efficacy of the PREVENT model in reducing hip fractures in LTC. Our hypothesis for the PREVENT trial can be found in the Discussion section: “*We hypothesize that an increased uptake of fracture prevention medications may translate to lower fracture rates in LTC; however, a large trial is needed to confirm the hypothesis.*” (page 16, lines 389-391)

4. The authors noted some existing models, such as the Bavarian Fall and Fracture Prevention model in Germany, and how such a model might not fit the Canadian system. It's important for the authors to also note whether the PREVENT model can be used outside of the Canadian health system and even if such a model is useful in all Canadian provinces.

The PREVENT model is an adaptation of the Bavarian Fall and Fracture Prevention model, but adapted for the province of Ontario, Canada. We have clarified this point in the discussion: “*Similar models to prevent fractures in LTC include the Bavarian Fall and Fracture Prevention model in Germany that focuses on exercise, documentation of falls, environmental adaptations, medication reviews, vitamin D, hip protectors, and education among healthcare providers (36,37). The PREVENT model is a version of the Bavarian Fall and Fracture Prevention model adapted for the Ontario, Canada healthcare system. Akin to the Bavarian model, our model uses similar components including exercise, fall prevention, environmental adaptations, medication reviews, hip protectors, and educational material and all homes receive one-on-one training on how to implement the model. The Bavarian Fall and Fracture Prevention study is one of the few large studies to implement an educational outreach program to implement fracture prevention guidelines into LTC (36,37). In the Bavarian study, the authors implemented a multifactorial educational outreach program and after one-year found an 18% reduction in hip fracture in residents from the intervention home (hazard rate ratio 0.82, 95% confidence interval 0.72-0.93) (36,37). While the model was effective, it was a non-randomized, quasi-experimental design that utilized insurance company staff to facilitate and disseminate knowledge on fall and fracture prevention. The PREVENT model used components of the Bavarian model to adapt the model to the Canadian billing and healthcare system and to reflect the differences between our healthcare systems. The next steps will be to conduct a large randomized controlled trial (NCT04947722) of the adapted PREVENT model in 122 LTC homes across Ontario, Canada to determine if the model reduces the rate of hip fractures in residents at high risk of fracture. The ultimate goal will be to implement the model across the country.*” (page 17, lines 408-429). The PREVENT model can be used in all Canadian provinces. Although we are not experts on the healthcare systems of other countries, it is our belief that the PREVENT model can be used internationally.

**Reviewer #2**: The research entitled “The iCARE feasibility non-experimental design study: An integrated collection of education modules for fall and fracture prevention for healthcare providers in long-term care” was conducted among healthcare providers of three long-term care (LTC) facilities in Ontario, Canada. The iCARE study was conducted to see the feasibility of implementing the PREVENT model to educate health workers working in LTC facilities on how to prevent falls and injuries in residents of LTC facilities. The manuscript is well-written and easy to follow. However, my great discomfort lies in the use of iCARE in the title of the paper. I have provided some input so that the paper can be made better.

1. The title says the study is about iCARE intervention. However, it has been explained very nominally in the paper. Instead, another intervention (PREVENT) has been described in much detail; however, iCARE should be explained in more detail. It is not clear how iCARE differs from PREVENT.

The iCare study is the feasibility study and project name, while PREVENT is the model that was implemented. To clarify your point, we have included the following sentence in the introduction: “*Before a trial can be implemented to determine the effectiveness of the PREVENT model in practice, we need to determine the feasibility of implementing the model under real-world conditions. The purpose of the iCare study was to determine the feasibility of implementing the PREVENT model (herein known as PREVENT) to healthcare providers in LTC. iCare” refers to the feasibility study and project name.*” (page 4, lines 91-95)

2. When reading the whole manuscript, the intervention was PREVENT, and iCARE was the project that administered PREVENT. Hence, I suggest that the authors make a distinction between these two things. If iCARE is a project, I believe explaining that in a study setting and removing it from the title would make more sense.

We have addressed this comment above (see response to Reviewer #2 Comment 1).

3. There is no result of the t-test in the result section. I recommend that the authors include bivariate results of the t-test, including t-statistics, p-value, and mean difference.

The t-test was removed from the results section, and we performed a McNemar test to assess knowledge uptake and the proportion of fracture prevention medication prescriptions. These findings have been added to the results section and are reported using a number, percentage and p-values, with aggregate results reported using absolute change. Results of the McNemar tests on knowledge uptake and related subset analysis can be found in the Knowledge Uptake section: “*We found that the educational module improved knowledge uptake of the LTC recommendations among healthcare providers. Knowledge uptake increased by 25% (pre = 57/108 = 52.8% versus post = 84/108 = 77.8%; p-value < 0.01). A knowledge uptake subset analysis was performed to assess healthcare providers knowledge surrounding FRS scores, corresponding fracture risk, administrating fracture prevention medications and supplements (calcium and Vitamin D), using hip protectors, and appropriate exercise. Results of this analysis indicate that knowledge uptake also increased by 25% (pre = 50/99 = 50.5% versus post = 75/99 = 75.8%; p-value < 0.01).*” (page 13, lines 308-315). Results of the audit report indicate: “*Home A results indicated the number and percentage of residents at high risk of fracture on fracture prevention medications increased by 16.5% (pre= 19/97 = 19.6% versus post= 35/97 = 36.1%; p-value = 0.005). Non-statistically significant results were found for the number of residents at high risk of fracture changed by 6.2% (pre = 52/97 = 53.6% versus post = 58/97 = 59.8%; p-value = 0.41) and the number and percentage of residents who sustained a fragility fracture in the last six months did not change (pre = 3/97 = 3.1% versus post = 3/97 = 3.1%; p-value = 1.0). Home B reported an absolute change of 3% (31% at baseline and 34% post-intervention), while Home C increased by 11% (26% at baseline and 37% post-intervention).*” (page 13, lines 313-320)

4. On page 13, line no. 312, Are you talking about “mean difference” instead of “mean?”

Findings have been updated to remove mean differences and these values were replaced with a number and percentage to report changes in knowledge uptake and fracture medication prescriptions and p-values to indicate statistical significance. These findings are referenced in the comment above (see response to Reviewer #2 Comment 3).

5. On page 14, line no. 316, authors have reported odds ratio. However, no regression techniques have been mentioned in the methods section. So, I am wondering how the authors got the odds ratio. Hence, I suggest that the authors review their quantitative data analysis techniques.

A regression analysis was not conducted in the iCare study. We have updated our Statistical Analysis section of the methods to reflect this and removed odds ratios and confidence intervals: “*We reported our recruitment rates and fidelity scores using descriptive statistics as a total score or value. Results of the focus groups were reported using content analysis and the primary author (IBR) coded the data and met to discuss and compare the codes with two reviewers (LK, LH) (32). Demographic characteristics of the local champion and leadership team were collected and reported using the number of individuals and percentages. To assess change in knowledge uptake, we conducted a McNemar test (using an exact significance (2-tailed)) for two related samples, for those who completed both pre- and post-assessments and reported the number and percentage of change in participants’ knowledge uptake. To report changes in fracture prevention medications, from the audit report, we conducted two data pulls at baseline and post-intervention; we included residents who “moved-in” (i.e., moved into the home during the post-intervention phase) in the analysis. We reported the number and percentage of change in fracture prevention medication prescriptions using a McNemar test. Quantitative results were analyzed using IBM SPSS Statistics for Windows, version 28 (Armonk, NY: IBM Corp) and qualitative transcripts were analyzed using NVivo, version 14 (QSR International Pty Ltd, Doncaster, VIC, Australia).*” (page 9, lines 199-211)

6. In the result section, it would be nice to see a joint display of some common variables in quantitative and qualitative data. For example, you can display a reduction in osteoporosis medication from QUANT and justify that from QUAL data.

The purpose of the study was to determine feasibility outcomes; jointly displaying common variables in quantitative and qualitative data is beyond the score of this paper; however, we will consider this for the PREVENT trial. The quantitative analysis was performed to assess change in knowledge uptake and fracture prevention medication prescriptions (see sections of manuscript referenced in response to Reviewer #2 Comment 3). The qualitative data we collected and analyzed evaluated the experiences of the local champions and leadership team throughout the study to describe their experiential accounts of the feasibility in implementing PREVENT in their home. These findings are reported under the Challenges, successes and adaptations: “*During the focus group, each home identified similar barriers to and facilitators of implementing PREVENT in practice. Challenges to implementing PREVENT included having an unclear sustainability strategy, misperceptions between fall and fracture prevention strategies, fear of prescribing fracture prevention medications, and time barriers with completing the audit report. During the focus group, the local champion expressed that it was unclear who would sustain the role of implementing PREVENT after the research team had left; but Home C suggested the falls program lead and/or staff educators should be involved upon study completion to sustain practices. The local champion cited lack of time and other commitments as a barrier to sustaining the model; all three champions noted that a major barrier was dedicating time to the audit and feedback report. To manually extract the data and link the variables to each resident for the audit report, the local champion required at least one full day per week for five weeks (i.e., 40 hours total). Moreover, several leadership members were unsure of the implementation plan after identifying a resident at high risk of fracture:*

*“I found myself often sometimes ignoring the prompts for FRS <Fracture Risk Scale> unless there was a specific reason. Cold calling families to say your fracture risk scale is high, and that’s the only reason I’m calling is weird.” [Home B]*

*Several healthcare professionals on the leadership team felt the strategy to prevent fractures (i.e., PREVENT) was akin to their current fall prevention strategy. It was perceived that if a healthcare professional could prevent a fall, it would translate to preventing a fracture.*

*“I think a lot of homes already are looking at all the PREVENT aspects, it’s just not known as PREVENT or they’re not grouping them together, but I think it’s just something that’s already being looked at from all the different disciplines.” [Home A]*

*Other healthcare providers believed PREVENT was a tool to ensure the staff were performing their fall prevention duties, rather than a model of change to prevent fractures in LTC.*

*“It’s worked well just as a tool to let us know if there’s any missing boxes that we’re not checking for fracture prevention. It’s very helpful with just making sure we’re looking at everything and yea, just crossing off any things we can possibly to do help the residents.” [Home A]*

*Additionally, several healthcare providers were unsure about the ethical implications of prescribing fracture prevention medications to residents at high risk of fracture. The main barrier to prescribing medications was fear of side effects (i.e., osteonecrosis of the jaw, atypical femur fractures, hypocalcaemia). Some providers were also concerned about the ethical implications of prescribing fracture prevention medications to residents who could not consent or had limited knowledge of the medication.*

*“We are worried that, we weren’t overly confident that would lead to prescription. So there was a trigger. We didn’t know if they were going to have informed consent and we didn’t know if our nursing staff knew enough about these medications to provide it” [Home B].*

*Successes: Despite some challenges, we also identified several facilitators to our model. Facilitators included easy access to the FRS, clear and succinct educational material catered to each healthcare professional, and an educational module with an accredited program for physicians and nurses. All three homes deemed it beneficial to have the educational module delivered in person. We also received positive feedback on the type of information included in our educational module. In particular, healthcare providers found it helpful to review real-world case studies followed by discussions and suggestions to overcome barriers to implementation (e.g., using hip protectors for residents with incontinence).*

*“A visual to see the walkers and those pieces, I think even the slide with the hip protectors, like how to apply them and which ones were incorrect. I think that’s a nice reminder because I didn’t even know how to position them correctly and I didn’t even have awareness of the FRS. Like I kind of knew like our FRAT score and our falls risk, and when I go to my units I know who the falls, the higher risk of falls are but I didn’t, and then I just thought of them like okay, we have hip protectors for them, like what are we doing about it but I didn’t necessarily do a full review of like let’s look at their meds and let’s look at their hips, are they wearing hip protectors, what are they, let’s look at all these other things. I wasn’t sort of looking at that.” [Home C].*

*Adaptations: The process of developing and implementing models into practice is an iterative process. Through this process, we recognized three adaptations to the PREVENT model including identifying additional characteristics for a successful local champion, developing an automated audit report, and creating tools to facilitate conversations for prescribing fracture prevention medications. It was suggested that the role of the local champion include co-championship with at least two healthcare professionals to help manage the implementation process. Suggested co-championship included one champion be either a registered nurse or a nurse practitioner and the second champion be a registered practical nurse or resident care manager. The benefit of including a resident care manager as a local champion is their established rapport with residents and caregivers, and their knowledge of daily practices and the types and frequency of assessments being done. Furthermore, the local champion must have a basic understanding of medications and have strong collaborations with other healthcare providers including physicians, nurses, dietitians, pharmacists, and physiotherapists. Additional co-champions may include a restorative care coach, RAI coordinator, and falls program lead. Home C described certain characteristics that may make a good local champion including someone who is interested and passionate about fracture prevention, willing to make change happen, and a good communicator. Other adaptations for future practice include working with the electronic medical record company to automate the audit report process, develop conversational toolkits (e.g., between nurses and residents) to aid dialogues about fracture prevention, and develop one-page educational guides for healthcare professionals and residents about the side effects of fracture prevention medications.*” (pages 11-12, lines 234-290). As the quantitative and qualitative variables were different and not part of our initial expected outcomes we reported them separately.

7. In the discussion section, I could not see the link between the Fidelity score and the knowledge score of long-term care medical staff. Is it possible to think in this direction as well?

The fidelity scores ranged from moderate to good indicating the local champions delivered the PREVENT model appropriately during the educational session; however, we did not conduct a specific analysis to see if there was a correlation between fidelity score and knowledge uptake as that was not one of our initial outcomes. We report them as separate outcomes: “*Our local champions successfully delivered PREVENT with a fidelity score ranging from moderate to good.”* (page 15, lines 357-358).

We indicated in the manuscript the following: *“We found that the educational module improved knowledge uptake of the LTC recommendations among healthcare providers. Knowledge uptake increased by 25% (pre = 57/108 = 52.8% versus post = 84/108 = 77.8%; p-value < 0.01). A knowledge uptake subset analysis was performed to assess healthcare providers knowledge surrounding FRS scores, corresponding fracture risk, administrating fracture prevention medications and supplements (calcium and Vitamin D), using hip protectors, and appropriate exercise. Results of this analysis indicate that knowledge uptake also increased by 25% (pre = 50/99 = 50.5% versus post = 75/99 = 75.8%; p-value < 0.01).*”  (page 13, lines 313-320)

**Reviewer #3**: The study evaluates the feasibility of implementing the PREVENT model, a multifactorial intervention to prevent fractures in long-term care (LTC) residents. Utilizing a mixed method strengthens the study. While the increase in osteoporosis medication prescriptions is a positive finding, the lack of statistically significant knowledge uptake seeks further investigation.

We conducted a McNemar test often applied in situations where you have a binary outcome (e.g., yes/no, success/failure) measured at two different times or under two different conditions for the same participant. As outlined in the results section of the manuscript, based on our updated findings from the McNemar tests, findings indicate a statistically significant increase in knowledge uptake. Statistical analyses that evaluated knowledge uptake, included assessment of knowledge regarding diet and supplements, exercise, hip protectors and medication prescriptions. Results indicate that knowledge uptake was statistically significant. We have modified the results section to include the following sentence: “*We found that the educational module improved knowledge uptake of the LTC recommendations among healthcare providers. Knowledge uptake increased by 25% (pre = 57/108 = 52.8% versus post = 84/108 = 77.8%; p-value < 0.01). A knowledge uptake subset analysis was performed to assess healthcare providers knowledge surrounding FRS scores, corresponding fracture risk, administrating fracture prevention medications and supplements (calcium and Vitamin D), using hip protectors, and appropriate exercise. Results of this analysis indicate that knowledge uptake also increased by 25% (pre = 50/99 = 50.5% versus post = 75/99 = 75.8%; p-value < 0.01).*” (page 13, lines 313-320)

**Reviewer #4**: Thank you for the opportunity to review this manuscript. The study is really a good initiative with the current increase in elderly/dependency. Anyway I have listed some comments to increase the quality of the manuscript.

1. Please avoid too much text within brackets.

We’ve reviewed the manuscript and removed unnecessary brackets.

2. better avoid the "We", "our"

Thank you for the comment. We wrote the manuscript in first person as it aligns with the recommendations of the NCBI Style Guide to improve clarity. In addition, the PLOS journals recommend writing in first person-voice.

3. Give emphasis on maintaining the flow of manuscript: Describe the results according to the stated objectives, then discussion for your results and finally conclusion. Please include some justifications for "no statistically significant increase" rather than simply stating proportions.

The results section was revised to indicate the order of the stated objectives: feasibility, challenges, successes and adaptations, knowledge uptake, and the audit report. Non-statistically significant findings were described in the Audit report section of the results with the associated p-values reported to provide evidence for the lack of statistical significance: “*Non-statistically significant results were found for the number of residents at high risk of fracture changed by 6.2% (pre = 52/97 = 53.6% versus post = 58/97 = 59.8%; p-value = 0.41) and the number and percentage of residents who sustained a fragility fracture in the last six months did not change (pre = 3/97 = 3.1% versus post = 3/97 = 3.1%; p-value = 1.0).*” (page 14, lines 336-342). Due to the short duration between pre- and post-measurements, we did not expect to see significant changes in the number of residents that sustained a fragility fracture, but measured this outcome to ensure that it was feasible to do so.

4. The discussion part of the manuscript need to be redrafted with some comparisons of previous similar studies

There are few studies on fall and fracture prevention models implemented in long term care homes. We included a section in the discussion that compares our results with non-LTC results: “*Similar models to prevent fractures in LTC include the Bavarian Fall and Fracture Prevention model in Germany that focuses on exercise, documentation of falls, environmental adaptations, medication reviews, vitamin D, hip protectors, and education among healthcare providers (36,37). The PREVENT model is a version of the Bavarian Fall and Fracture Prevention model adapted for the Ontario, Canada healthcare system. Akin to the Bavarian model, our model uses similar components including exercise, fall prevention, environmental adaptations, medication reviews, hip protectors, and educational material and all homes receive one-on-one training on how to implement the model. The Bavarian Fall and Fracture Prevention study is one of the few large studies to implement an educational outreach program to implement fracture prevention guidelines into LTC (36,37). In the Bavarian study, the authors implemented a multifactorial educational outreach program and after one-year found an 18% reduction in hip fracture in residents from the intervention home (hazard rate ratio 0.82, 95% confidence interval 0.72-0.93) (36,37). While the model was effective, it was a non-randomized, quasi-experimental design that utilized insurance company staff to facilitate and disseminate knowledge on fall and fracture prevention. The PREVENT model used components of the Bavarian model to adapt the model to the Canada billing and healthcare system and reflect the differences between our healthcare systems. The next steps will be to conduct a large randomized controlled trial (NCT04947722) of the adapted PREVENT model in 122 LTC homes across Ontario, Canada to determine if the model reduces the rate of hip fractures in residents at high risk of fracture. The ultimate goal will be to implement the model across the country. We hypothesize that an increased uptake of fracture prevention medications may translate to lower fracture rates in LTC; however, a large trial is needed to confirm the hypothesis*.” (pages 17, lines 408-429).

**Reviewer #5**: This study is apt! It is aligning with a contemporary prevention algorithm in the area of reducing falls especially among elderly. This study has public health significance and implications. The tools used for the purpose of training and data collections are in tandem with the title and study protocols. The findings are also evidenced-based. The Model adopted actually fit the study. The outcome of the study adds to the existing body of knowledge and will also serve as a further benchmark for other studies.

Thank you for your feedback and comments.

**Reviewer #6**: The paper was interesting and well described regarding the feasibility study of implementing an intervention. However, it would be good to additionally explain the relation of iCARE and PREVENT.

1. In result section, it would be better understandable if the recruitment outcomes (time to recruit LTC homes, time consumed for recruiting local champion, and time spent for deliver knowledge to care team, time used for providing audit and feedback repot) were explicitly demonstrated in table. Eventually, the explanation of getting to outcomes according to the using framework should be done. Moreover, the mechanism of change in health care provider behavior according to PREVENT model should also be elaborated.

The feasibility outcome results listed above have been inputted into a table in the Supplemental Material section (S4 Table). This table is provided below in response to this comment. Changes in healthcare provider behaviour have been integrated in the Knowledge Uptake section of the results: “*Healthcare professional in Home B were prompted by the PREVENT model to develop and implement a “Fracture Prevention in LTC Order Set” as a multidisciplinary approach to care. Physicians, nurse practitioners, physiotherapists and dietitians used this order set at the time of study to review criteria for high fracture risk and non-pharmacological and pharmacological medical orders for residents living in their home. Furthermore, use of the order set will be rolled out to nurses who were identified as a key facilitator to integrate this tool into practice across the home. Changes in healthcare provider behavior and practice were also observed in Home C where providers reported greater awareness of the fracture risk scale and residents' fracture risk. They also shared that their pharmacist now reviews fracture risk during quarterly assessments and implemented a formal policy for hip protectors, which was previously lacking. Additionally, this home integrated the FRS Manual Calculation Tool into their admission process, ensuring that residents receive a fracture risk score upon admission.*” (pages 13-14, lines 321-332).

**S4 Table: Feasibility outcomes results**

| Recruitment rate (organizational level – length of time to recruit three LTC homes) | 5 months |
| --- | --- |
| Recruitment rate (participant level - length of time to identify local champion) | 1 month |
| Recruitment rate (participant level - length of time for local champion to review educational materials, adapt material to the LTC home, and develop audit and feedback report) | 2 months |
| Recruitment rate (summary - length of time to recruit a LTC home, train the local champion and implement the PREVENT model) | 10 to 12 months |

2. Regarding to secondary outcomes, please explain why there was no change in knowledge uptake but have change on prescription of medication. Should there be any other secondary outcomes regarding diet, exercise, environmental adaptation, and hip protector adoption.

As part the revisions made in response to this review, we conducted a test using the McNemar test often applied in situations where you have a binary outcome (e.g., yes/no, success/failure) measured at two different times or under two different conditions for the same subjects. As outlined in the results section of the manuscript, based on our updated findings from the McNemar tests, findings indicate a statistically significant increase in knowledge uptake. Statistical analyses that evaluated knowledge uptake, included assessment of knowledge regarding diet and supplements, exercise, hip protectors and medication prescriptions. Additionally, we did not collect data on adopting secondary outcomes, but for the larger PREVENT trial we intend to conduct we will collect these outcomes. We have included the restricted number of secondary outcomes as a limitation to our study with the goal of collecting them in a future study. “*Given that our intervention was multifaceted, it was challenging to identify the most significant components (e.g., educational outreach, local champion, audit and feedback report) that would result in effective implementation of PREVENT. Moreover, measures of organizational context such as work culture and leadership style, which could have a significant impact on our model’s components, were not captured in the iCARE study. For this study, we did not involve residents or their caregivers during the implementation process; however, the Family Councils Ontario and Ontario Association of Residents' Councils, which represent resident and family perspectives, advised upon the 2015 guidelines and the development of the PREVENT model. Future adaptations for the PREVENT model will include end-users on the research team and a Resident Advisory Committee will be formed to obtain resident feedback on study-related materials to aid the implementation process. Lastly, we did not collect secondary outcomes including changes in diet, exercise, environmental adaptations, and hip protector adoption due to the limited resources in the iCARE study. For the larger PREVENT trial, we will collect these secondary outcomes to gain a larger perspective of the changes the PREVENT model may provide.*” (page 18, lines 454-468)

3. Please provide further discussion on the relation of fear on side effects of medication and increasing of medication prescription.

We have included a paragraph on the fear of side effects of medications and a potential solution to increase medication prescribing: “*Although we found a 7% increase in the number of prescriptions before and after the intervention; healthcare providers and residents expressed concerns with prescribing and taking fracture prevention medications, respectively. Physicians were concerned about polypharmacy and the potential effects of residents who are taking multiple medications, while residents were more concerned about possible drug-related side effects than the consequences of fractures. Fear of osteoporosis drugs is a common concern among people living with osteoporosis. A survey study of 853 individuals, conducted by the National Osteoporosis Foundation found that 38% of respondents had been prescribed a medication but refused to take it, with 79% stating that fear of side effects was the reason for refusing the medication (35). Approximately 43% of survey respondents thought that the risk of side effects with osteoporosis treatment was greater than the benefit. Implementing models with behaviour change techniques will be important to target residents specifically to address their fears and concerns. Potential adaptations to the PREVENT model may include targeting residents directly, in addition to healthcare workers, through an educational outreach program on the risks of fractures versus the benefits of taking an osteoporosis medication. The outreach program should be delivered by a potential resident council member that has developed rapport with residents in the LTC home.*” (page 16, lines 392-407)

**Reviewer #7**: Firstly, I would like to highlight the great value of the study's aim and results. The technical standard of the approach appears to be excellent; it thoroughly describes the details of the iCARE study, PREVENT, and their barriers and facilitators.

1. The publication holds significant value, and to continue fostering transparency, collaboration, and innovation, I strongly recommend making the dataset and codes available on a repository. This action does not prevent authors from sharing data upon request. However, sharing not only enhances the credibility and reproducibility of research but also accelerates scientific progress by enabling interdisciplinary collaboration and new discoveries.

We agree that sharing the data is important to accelerate scientific progress and new discoveries. The deidentified data for replication can be found in the S6 File (S6 File: Deidentified data for replication). We have also included a supplementary Table (S5 Table Full list of themes and codes), which is shown below.

**S5: Full list of themes arising from the content analysis. Themes are shown with illustrative quotes.**

| **Themes** | **Quote** |
| --- | --- |
| **Challenges**   - Unclear sustainability strategy - Misperceptions between fall and fracture prevention strategies - Fear of prescribing osteoporosis medications - Time barriers with completing the audit report | “I found myself often sometimes ignoring the prompts for FRS unless there was a specific reason. Cold calling families to say your fracture risk scale is high, and that’s the only reason I’m calling is weird.” [Home B]  “I think a lot of homes already are looking at all the PREVENT aspects, it’s just not known as PREVENT or they’re not grouping them together, but I think it’s just something that’s already being looked at from all the different disciplines.” [Home A]  “It’s worked well just as a tool to let us know if there’s any missing boxes that we’re not checking for fracture prevention. It’s very helpful with just making sure we’re looking at everything and yea, just crossing off any things we can possibly to do help the residents.” [Home A]  “We are worried that, we weren’t overly confident that would lead to prescription. So, there was a trigger. We didn’t know if they were going to have informed consent and we didn’t know if our nursing staff knew enough about these medications to provide it” [Home B]. |
| **Facilitating Factors**   - Easy access to the FRS - Clear and succinct educational material tailored to each healthcare professional - Educational module with an accredited program for physicians and nurses | “A visual to see the walkers and those pieces, I think even the slide with the hip protectors, like how to apply them and which ones were incorrect. I think that’s a nice reminder because I didn’t even know how to position them correctly and I didn’t even have awareness of the FRS. Like I kind of knew like our FRATscore and our falls risk, and when I go to my units I know who the falls, the higher risk of falls are but I didn’t, and then I just thought of them like okay, we have hip protectors for them, like what are we doing about it but I didn’t necessarily do a full review of like let’s look at their meds and let’s look at their hips, are they wearing hip protectors, what are they, let’s look at all these other things. I wasn’t sort of looking at that.” [Home C]. |
| **Adaptations**   - Identifying additional characteristics for a successful local champion - Developing an automated audit report - Creating tools to facilitate conversations for prescribing osteoporosis medications | “… once everyone gets used to that new documentation I think they will adjust to the fact that we have an FRS and also a FRAT because resident care managers are mostly concerned about the FRAT score whereas now that we’ve just changed the falls, then the nurses that are completing those assessments will be focused on both instead of just the one.” [Home B] |

FRS = Fracture Risk Scale; FRAT = Falls Risk Assessment Tool

2. By the end of the discussion, authors do recognize that residents or family caregivers were not considered during the implementation process. I believe this limitation extends beyond the implementation process. Residents' experiences and opinions— their voices—should be considered from the planning of the approach and study through all steps. Particularly in LTC, where it is common to neglect the autonomy of people who live in these facilities, a program developed to prevent an event as common as a fall among them needs to consider the impacted people. Their experiences should also inform the design and serve as a source of evaluation about the approach.

We fully agree with the reviewer on the importance of the involvement of residents and family caregivers in co-designing research. We have included a paragraph outlining how the experiences and voices of residents’ living in LTC will be considered in the larger randomized control trial – the PREVENT trial: “*For this study, we did not involve residents or their caregivers during the implementation process; however, the Family Councils Ontario and Ontario Association of Residents' Councils advised upon the 2015 guidelines and the development of the PREVENT model. Future adaptations for the PREVENT model will be to include end-users on the research team and have LTC residents join a Resident Advisory Committee to review and provide feedback on study-related materials to aid the implementation process.*” (page 18, lines 459-464)

3. For instance, in the third step of PREVENT, when the local champion presents the adapted material during the leadership team meeting with various professionals, some residents could have been included. I understand that the involvement of LTC residents in advocating for their needs depends on the management model adopted by the facility. However, as research takes place, it is a responsibility to promote opportunities for an integrated approach to legitimize people's autonomy, as PREVENT's goal is to prevent falls and fractures among LTC residents. It's only understandable that they are also involved.

Thank you for your feedback and comments. We agree with these recommendations. As the iCare study was a feasibility study and did not include residents or their caregivers during the implementation process, future adaptations to PREVENT trial that will include a Resident Advisory Committee: “*Future adaptations for the PREVENT model will be to include end-users on the research team and have LTC residents join a Resident Advisory Committee to review and provide feedback on study-related materials to aid the implementation process*.” (page 18, lines 459-464)

4. One of the goals of the iCare study centered on training trainers, specifically the local champion, which is a great strategy. I recommend sharing more details on the motivation, challenges, and strategies for selecting the local champion and their experience on this role. Understanding this role's experience seems to be a key component for the sustainability of the approach in LTC.

Local champions identified having to allocate specific periods of time to complete the audit report as this component of the iCare study was time consuming and acted as a barrier to completing other daily tasks within their role in LTC. Challenges and successes of local champions and the leadership team can be found in the Challenges, successes and adaptations section of the manuscript and are provided in the response to Reviewer #2 Comment 6. We included the following paragraph to describe the different healthcare professionals and characteristics that local champions or members of the leadership advised may make a good local champion in the future: “*The benefit of including a resident care manager as a local champion is their established rapport with residents and caregivers, and their knowledge of daily practices and the types and frequency of assessments being done. Furthermore, the local champion must have a basic understanding of medications and have strong collaborations with other healthcare providers including physicians, nurses, dietitians, pharmacists, and physiotherapists. Additional co-champions may include a restorative care coach, RAI coordinator, and falls program lead. Home C described certain characteristics that may make a good local champion including someone who is interested and passionate about fracture prevention, willing to make change happen, and a good communicator. Other adaptations for future practice include working with the electronic medical record company to automate the audit report process, develop conversational toolkits (e.g., between nurses and residents) to aid dialogues about fracture prevention, and develop one-page educational guides for healthcare professionals and residents about the side effects of fracture prevention medications*.” (page 13, lines 299-311)

5. The manuscript reports absolute changes in outcomes, which raises questions about statistical rigor, particularly concerning sample sizes of certain groups. I recommend using appropriate statistical tests to compare differences between groups, especially when dealing with smaller sample sizes.

We revised our statistical methods to include the McNemar test to determine whether there are differences on a dichotomous dependent variable between two related groups. Please see the response to Reviewer #2 Comment 5 describing the updated statistical analysis methods section. A total of 748 LTC residents’ pre-intervention and 745 residents’ post-intervention audit report data were analyzed across the three participating LTC homes (page 14, table 4 – see below).

**Table 4: Aggregated pre-and-postaudit report**

|  | **Homes A, B and C** | |
| --- | --- | --- |
|  | **Pre-intervention** | **Post-intervention** |
| Total number of residents in Home A, B, and C | 748 | 745 |
| # high fracture risk residents (%) | 430 (57%) | 433 (58%) |
| # low fracture risk residents (%) | 315 (42%) | 306 (41%) |
| # of residents that sustained a fragility fracture (%) | 22 (3%) | 28 (4%) |
| # high fracture risk residents on fracture medications (%) | 122 (28%) | 153 (35%) |

Note: The number of residents pre-and-post intervention are different because of some residents “moved-in” (i.e., moved into the LTC home) and “moved-out” (i.e., passed away) during the study time period.

6. I agree with the authors that effective and sustainable knowledge translation models are essential to encourage the uptake of evidence-based practices, and that it takes time. However, I would like to share some concerns if a randomized controlled trial to implement PREVENT will take place in 122 homes. The limitations presented in this paper will need to be addressed, such as: i) using an automated system to monitor and evaluate the educational impact; ii) addressing the potential flaw in sustaining the program's success on medication prescription when polypharmacy is a big concern in LTC. Perhaps a multivariate analysis could be conducted to include other aspects of a person's eligibility to have osteoporosis medication prescribed; and as mentioned, iii) involving residents from the planning of the approach and study through all steps, including monitoring and evaluation.

Thank you for the suggestions. We will consider your points for our next study when we conduct the PREVENT randomized controlled trial.
